# Noninvasive DBS-Based Approaches to Assist Clinical Diagnosis and Treatment Monitoring of Gaucher Disease

**DOI:** 10.3390/biomedicines11102672

**Published:** 2023-09-29

**Authors:** Claudia Rossi, Rossella Ferrante, Silvia Valentinuzzi, Mirco Zucchelli, Carlotta Buccolini, Sara Di Rado, Daniela Trotta, Liborio Stuppia, Luca Federici, Maurizio Aricò

**Affiliations:** 1 Center for Advanced Studies and Technology (CAST), “G. d’Annunzio” University of Chieti-Pescara, 66100 Chieti, Italy; claudia.rossi@unich.it (C.R.); rossella.ferrante@unich.it (R.F.); silvia.valentinuzzi@unich.it (S.V.); m.zucchelli@unich.it (M.Z.); carlottabuccolini@alice.it (C.B.); sara.dirado12@gmail.com (S.D.R.); stuppia@unich.it (L.S.); luca.federici@unich.it (L.F.); 2 Department of Innovative Technologies in Medicine and Dentistry, “G. d’Annunzio” University of Chieti-Pescara, 66100 Chieti, Italy; 3 Department of Pediatrics, S. Spirito Hospital, Azienda Sanitaria Pescara, 65121 Pescara, Italy; daniela.trotta@asl.pe.it; 4 Department of Psychological, Health and Territorial Sciences, School of Medicine and Health Sciences, “G. d’Annunzio” University of Chieti-Pescara, 66100 Chieti, Italy

**Keywords:** lysosomal storage diseases, Gaucher disease, newborn screening, mass spectrometry

## Abstract

Gaucher disease (GD) is an autosomal recessive inborn error of metabolism, belonging to the group of lysosomal storage diseases (LSDs). GD is caused by a defect in lysosomal glucocerebrosidase, responsible for glucosylceramide breakdown into glucose and ceramide. Because of this dysfunction, glucosylceramide progressively accumulates in the liver, spleen, bone marrow, bones, and in other tissues and organs, also causing anemia, hepatosplenomegaly, thrombocytopenia, and bone symptoms. Depending on neurological symptoms, GD is classified into three main types. Treatment options for LSDs, including enzyme replacement therapy, hematopoietic stem cell transplantation, small molecular weight pharmacologic chaperones, and, for some LSDs, gene therapy, are increasingly available. For this reason, many efforts are aimed at implementing newborn screening for LSDs since early detection accompanied by a prompt intervention has been demonstrated to be essential for reducing morbidity and mortality and for improved clinical outcomes. Herein, we report two siblings of preschool age, presenting with hepatosplenomegaly and thrombocytopenia. The initial suspicion of GD based on the clinical picture was further supported by biochemical confirmation, through newborn screening workflow, including first- and second-level testing on the same dried blood spot samples, and finally by molecular testing.

## 1. Introduction

Gaucher disease (GD) is a lysosomal storage disease (LSD) affecting the recycling of cellular glycolipids. Glucocerebroside is ordinarily degraded to glucose and lipid components, while it accumulates within the lysosomes of cells in GD. The incidence of GD in the general population ranges between 0.4 and 5.8 per 100,000 births, with a prevalence of 0.7 to 1.8 per 100,000 [1]. The non-neuronopathic form of the disease, type 1 (GD1) accounts for 90 percent of patients. GD1 is the most common type seen in the Ashkenazi Jewish population. The incidence rate of GD1 in non-Jewish populations ranges approximately between 1 in 40,000 [2] and 1 in 86,000 [3] livebirths. The incidence rate of neuronopathic GD (GD2) is about 1 in 150,000 [4]. The incidence rate of GD3 (subacute or chronic neuronopathic GD) is 1 in 200,000, with a much higher prevalence than GD2 due to longer survival. GD3 is widely distributed in many areas including Northern Europe, Eastern Asia, and Egypt [5].

Despite attempts to categorize the three types of GD, they comprise a continuum from severely affected collodion babies through those with acute and chronic neuronopathic forms, to patients with non-neuronopathic GD manifesting with skeletal and visceral involvement or Parkinson disease, to older adults with mild or no clinical manifestations [6,7].

GD results from deficiency of lysosomal glucocerebrosidase (GBA, also known as glucosylceramidase or acid beta-glucosidase) [6,7], a glycoprotein enzyme whose major substrate is glucocerebroside, a component of the cell membrane that is distributed widely in many organs.

The deficiency of GBA leads to the accumulation of glucocerebroside and other glycolipids within the lysosomes of macrophages [6]. The tissue levels of these compounds may be increased 20 to 100 times [8]. 

Macrophages stuffed with glycolipids accumulate in many tissues, including those of the liver, spleen, bone, and bone marrow. However, pathologic lipid accumulation in macrophages accounts for less than 2 percent of the additional tissue mass in the liver and spleen [9]. The inflammatory and hyperplastic cellular response accounts for the additional increase in organ weight and volume [10]. 

The pathologic processes also occurring within bone are different: decreased mineral density, marrow infiltration, and infarction of bone. Although uncertain, the mechanisms leading to decreased bone mineral density may involve a failure to achieve peak bone mass, abnormal osteoclast regulation, or overproduction of cytokines by activated macrophages [11]. In an in vitro model of GD, primitive hematopoiesis and proliferation of mesenchymal progenitors were impaired, suggesting that cytopenias are caused by an intrinsic defect in addition to hypersplenism and bone marrow infiltration with Gaucher cells [12]. 

We describe the presenting features, diagnostic approach, and initial response to therapy in two siblings with GD. In particular, the present study reports on the familial case of two siblings of preschool age presenting with hepatosplenomegaly and cytopenia, leading to referral of case 1 for suspected acute leukemia. After ruling out leukemia, GD became the clinical diagnosis, to be supported by biochemical confirmation, through newborn screening workflow, revealing the significantly low glucocerebrosidase activity and the strong accumulation of glucosyl-sphingosine on the same dried blood spot samples, and finally by molecular testing.

## 2. Case Presentation

### 2.1. Clinical Presentation

#### 2.1.1. Family History

Healthy parents of Moroccan origin, reporting themselves as unrelated; the mother was gravida 5, para 5. No evidence of familial diseases.

#### 2.1.2. Case 1

The index case is a 22-month male, born at term after a regular pregnancy, with an uneventful neonatal period. He did well until he was 20 months old, when abdominal distension was noted, followed by progressive asthenia. Thus, he was first seen at the local hospital where severe anemia was documented. The child was referred under the suspicion of acute leukemia.

On admission, he was pale, with normal skin and no evidence of hemorrhagic lesions. He had a weight of 11 Kg (25–50th centile); length of 84 cm (10–25th centile); and head circumference of 43 cm (<5th centile). His respiratory activity was normal, while the abdomen was prominent with marked hepatosplenomegaly. No lymphadenomegaly and no bone pain were apparent. The child was barely able to walk, but his neurological examination was normal. The main presenting features are summarized in Table 1.

**Figure 1:**
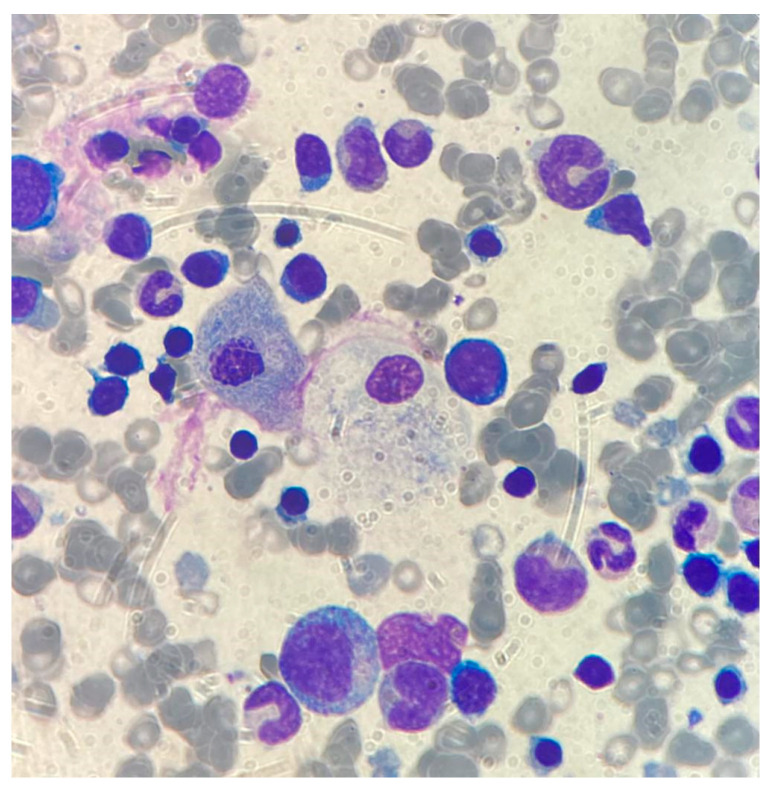
Bone marrow aspirate. MGG, 100×. Large monocytes engulfed with storage material.

In order to rule out leukemia or other hemo-proliferative disorders, bone marrow aspirate was performed: while leukemic metaplasia was not present, large macrophages stuffed with storage material were detected at morphological examination (Figure 1).

Thus, within a couple of hours, leukemia was ruled out and the diagnosis of possible GD was made; confirmation by biochemical study was requested on the same day. 

#### 2.1.3. Case 2

Following confirmed diagnosis in the index case, we asked detailed information of the remaining four siblings. The older, 41-month sister, (case 2) was reported as well doing. Yet, on physical examination, she was pale, with normal skin and no evidence of hemorrhagic lesions. She had a weight of 14.9 kg (54th centile); height of 92 cm (11th centile); and head circumference of 50 cm (78th centile). Her respiratory activity was normal, while the abdomen was prominent with hepatosplenomegaly. No lymphadenomegaly, no bone pain, and normal neurological examination were reported. Her main presenting features are summarized in Table 1. Based on his brother diagnosis, bone marrow aspirate to rule out leukemia was considered not necessary since she was considered very likely affected by GD as a familial disease, and confirmation by biochemical study was requested. 

### 2.2. Newborn Screening Workflow: First and Second-Level Testing

Newborn screening (NBS) for LSDs is performed as a first-tier test by measurement of lysosomal enzymatic activities in dried blood spot (DBS) samples. More precisely, there are two currently available methodologies used for determination of enzymatic activities: tandem mass spectrometry (MS/MS) and digital microfluidics fluorimetry (DMF-F). Multiplexing of the LSD enzymes are guaranteed by both platforms; the MS/MS has a higher analytical precision than DMF-F [13]. DBS samples are collected within 48–72 h after birth, by heel-pricking and drying of capillary blood onto filter paper. Details of the workflow adopted for primary screening of GD, FD, and MPS-I by FIA-MS/MS, as well as information on second-tier testing for the quantification of LysoGb1 and LysoGb3 by LC-MS/MS [14,15], are fully described in the Supplementary Materials.

Herein, the entire diagnostic workflow for NBS was applied on the DBS samples from pediatric patients with clinical diagnosis of GD.

### 2.3. Application of NBS Primary Screening and Second-Tier Testing on the Suspected Pediatric Patients and Relatives

Case 1. First DBS sample, collected at 22 months of age, underwent primary screening, revealing low GBA activity (0.385 μmol/L/h; cut-off > 3.89 μmol/L/h). Through workflow, reduced enzymatic activity indicated second-tier testing on DBS, which confirmed the clinical diagnosis of GD with the specific biomarker LysoGb1 = 662.63 nM (cut-off < 31.1 nM). 

Case 2. First DBS sample from the older sister, collected at 41 months of age, underwent primary screening, and showed low GBA activity (0.647 μmol/L/h; cut-off > 3.89 μmol/L/h). Accordingly, the second-tier testing on DBS confirmed the clinical diagnosis of GD with LysoGb1 = 616.49 (cut-off < 31.1 nM). 

A familial study was carried out in all available members (i.e., mother, father, two of the three other siblings) for the phenotypic profile by quantifying both GBA activity and LysoGb1 levels, which exhibited normal values. 

### 2.4. Molecular Confirmation Testing by Sanger Sequencing

**Figure 2:**
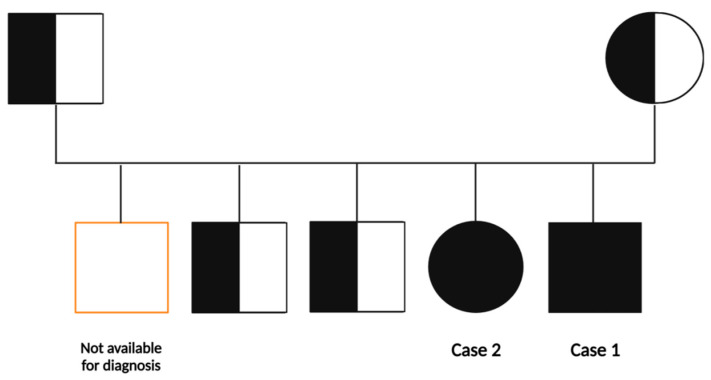
Pedigree of the Moroccan family with Gaucher disease, due to c.1448T>C (p.Leu483Pro) variant in *GBA1* gene.

The entire family was invited for mutation analysis of the gene *GBA1*, associated with GD (Figure 2). Sanger sequencing of the *GBA1* gene (NM_ 000157.4) evidenced the pathogenic variant c.1448T>C (p.Leu483Pro). The c.1448T>C variant causes the thymine nucleotide to be changed into a Cytosine nucleotide at position 1448 in exon 10 of the *GBA1* gene, causing the substitution of the amino acid Leucine by the amino acid Proline at codon 483 of the encoded protein (p.Leu483Pro). The molecular testing revealed the pathogenic familial mutation on both alleles for the symptomatic siblings. The familial mutation was found in monoallelic (heterozygous) form in both asymptomatic parents and the two older siblings, aged 18 and 13 years, respectively, also asymptomatic. One asymptomatic older sibling was not available for the diagnostic study. Sanger sequencing is described in full detail in the Supplementary Materials, with Figure S2 for the sequencing chromatographs showing the mutation of the *GBA1* gene in both GD cases as well as in the healthy carrier collaterals.

## 3. Diagnosis, Treatment and Follow-Up of the Patients

Following confirmation of the diagnosis of GD for both siblings, disease staging for assessment of possible bone lesions and CNS involvement was carried out with negative results. Based on the combination of clinical features, i.e., hepatosplenomegaly and cytopenia, with no evidence of bone or CNS involvement, these children were recognized as belonging to Type 1 (GD1; OMIM # 230800), the non-neuronopathic variant, with prevalent involvement of the liver, spleen, bone, and hematological system.

The enzyme replacement therapy (ERT) by i.v. imiglucerase (72 U/kg every other week) was started for the little boy (case 1). Soon thereafter, ERT by i.v. imiglucerase (66 U/kg every other week) was also started for his older sister (case 2). Treatment was very well tolerated and is still ongoing, after 12 and 7 doses administered, respectively, both at the dose of 60 U/kg. 

Clinical response was soon evident in both kids, with progressive reduction of the abdominal circumference and regression of cytopenia. The parents reported that the young boy (case 1), who was barely able to stand and walk without help at the time of the diagnosis, progressively improved his motor ability up to being able to run. 

Both patients had a reduction of liver and spleen size during ERT: liver diameter reduced in case 1 from 13 to 9.5 cm and in case 2 from 11 to 9 cm. Spleen size reduced as well: case 1 had a spleen length of 16 cm at the diagnosis, while at last follow-up the spleen dimensions were 11 × 3.7 × 3.5 cm; case 2 had an initial spleen length of 15 cm, while at last follow-up the spleen dimensions were 11 × 4.2 × 2.8 cm.

**Figure 3:**
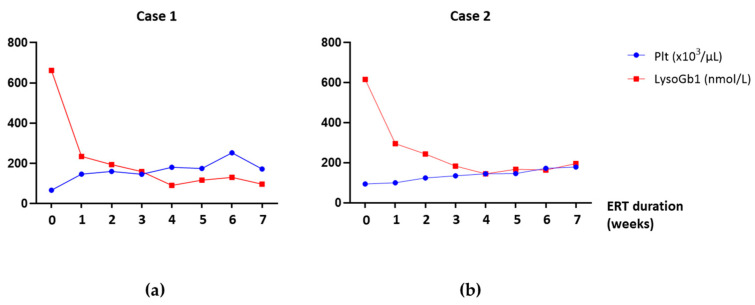
Multiparameter evaluation of response to therapy. LysoGb1 levels in DBS and peripheral blood platelet counts, before (time point 0) and after fortnightly ERT administrations, in two children with GD1, case 1 (**a**) and case 2 (**b**). In the graph, the value for platelet count is to be considered as x 10^3^/μL.

Figure 3 shows the trend of LysoGb1 levels quantified by second-tier testing on DBS samples as well as platelet count during the course of ERT, parameters monitored every 2 weeks at the time of the therapeutic session. As represented in Figure 3, a marked reduction of the accumulated biomarker in both patients was already observed during the first ERT administrations, with LysoGb1 levels reaching stability after the first four treatments. Moreover, the platelet count improved gradually during therapy, finally reaching normal values.

Interestingly, a good and significant correlation between the two detected parameters (LysoGb1 levels and platelet count) in case 1 (Pearson correlation coefficient r =−0.80; *p*-value = 0.0170) and case 2 (Pearson correlation coefficient r = −0.73; *p*-value = 0.0147) were found. In consideration of the presenting features at diagnosis and the improvement in clinical evidence during ERT for case 1, the correlation between the abdominal circumference and LysoGb1 levels was also investigated, showing a strong correlation between the two factors (Pearson correlation coefficient r = 0.96; *p*-value = 0.001) (Figure S3 of Supplementary Materials). Details of correlation analysis are described in the Supplementary Materials.

## 4. Discussion

The present study describes a sibling pair of preschool age, diagnosed with GD based on the clinical manifestation of splenomegaly and cytopenia. Bone marrow aspiration, performed to rule out possible acute leukemia, clearly showed at morphological evaluation typical storage cells, which oriented the diagnosis. Children with splenomegaly and cytopenia should be rapidly addressed with a diagnostic work-up for GD. In the recent GAU-PED study, Pession et al. report that 14 out of 154 patients considered at high risk for GD because of cytopenia and organomegaly were diagnosed with confirmed GD [16]. 

Based on the combination of clinical features, i.e., hepatosplenomegaly and cytopenia, with no evidence of bone or CNS involvement, these children were recognized as belonging to Type 1 (GD1; OMIM # 230800), the non-neuronopathic variant, with prevalent involvement of the liver, spleen, bone, and hematological system.

Interestingly, the parents did not realize that the slightly older sister was also affected, despite a very similar phenotype, so that the diagnosis was made only upon our specific request to check the remaining family members. As described above, GD is an autosomal recessive inborn error of metabolism resulting from biallelic mutations in the *GBA* gene on chromosome 1q21 [17] and causes a dysfunction of GBA in lysosomes [7]. This lysosomal enzyme is responsible for the breakdown of glucosylceramide into glucose and ceramide. Because of the enzymatic defect, affected individuals are unable to metabolize the specific substrate, glucosylceramide, leading to its progressive accumulation in the lysosomes of the liver, spleen, bone marrow, bone, and of other tissues and organs. Thus, affected patients exhibit widespread symptoms including anemia, hepatosplenomegaly, thrombocytopenia, and bone symptoms [18,19]. Accumulation of glucosyl-sphingosine (the deacylated form of glucosylceramide) in the brain is thought to underlie the development of neurological symptoms in GD [19,20]. 

Thus, following clinical diagnosis, the non-routinary biochemical work-up through the NBS workflow was started to document the defect in GBA activity and then confirmation was achieved by mutation analysis [21]. Even though the standard biochemical diagnosis of GD involves demonstrating GBA deficiency in blood leukocytes or cultured skin fibroblasts, the use of DBS samples for the enzymatic measurement only recently became a valid alternative for the rapid diagnosis of GD as well as for other LSDs [22]. Nowadays, the advantages offered by the use of DBS samples are well-recognized and include small blood volume, easy sample collection (far less invasive than venipuncture), and easier and less expensive storage and shipping [23]. 

After definitive diagnosis of GD1, and after both of the siblings had started ERT, a follow-up was conducted by closely monitoring different clinical and biochemical parameters, as a multiparameter evaluation of the response to therapy. The careful follow-up highlighted the potential value of LysoGb1: indeed, LysoGb1 level decrease was well correlated with clinical improvement during ERT. This evidence suggests that DBS-based LysoGb1 determination is a highly valid and noninvasive approach for therapeutic monitoring of GD patients, of importance in the evaluation of ERT efficacy [24,25,26].

While LysoGb1 as a biomarker of GD disease has already been recognized, we have shown here that its values is well correlated with the improvement of clinical parameters during treatment. Above all, we have shown that it is both possible and convenient to monitor LysoGb1 via the analysis of DBS rather than venipuncture, a much-preferred option by patients, especially those of pediatric age [27].

Through family history we investigated possible parental consanguinity, which was strongly denied. Yet, careful evaluation of their geographical origin showed that they originated from little villages sharing an obligate route, which suggested a likely common origin. Haplotype analysis confirmed that the parents not only share the same pathogenic mutation, but also share the entire haplotype, thus confirming their very likely consanguinity.

It is well recognized that GD is one of the most prevalent of the LSDs, a large group of inherited metabolic diseases that globally present a combined frequency of ~1:5000 live births, even if the real prevalence might be higher than estimated when considering undiagnosed patients [28]. Moreover, a global evaluation of GD incidence and prevalence identified important deficiencies in data for specific countries such as for Africa, India, and China [29]. Anyway, Nalysnyk et al. estimated the incidence of GD in the general population between 0.39 and 5.80 per 100,000 live births [1]. More interestingly, a study reported that GD is not rare in Morocco, and type 1 is the most common. This is due to the high frequency of consanguineous marriages [30].

The c.1448T>C variant found in this family is one of the four most common pathogenic variants for GD accounting for about 90% of the pathogenic variants; the frequency of this homozygous genotype in the affected patients’ population is 6% [31].

In their pioneer genotype–phenotype study of GD1, Zimran et al. had already detected four mutations in the DNA of 47 unrelated patients with type GD1 [32]. Two of the mutations, 1226 and 1448, and a new mutation (XOVR) representing cross-over between the *GBA* gene and its closely linked pseudogene, were found. Based on their findings, mutation 1226 was associated with a mild clinical phenotype, while mutation 1448 was associated with a more severe phenotype. In the present case, homozygosity for the so-called 1448 mutation, now defined as c.1448T>C (p.Leu483Pro), confirms its pathogenic role in causing a “chronic” phenotype of GD1, which may be included in type B, with no evidence of bone or CNS involvement during the first two or three years of life [32]. This variant involves a non-conservative amino acid change in the Glycosyl hydrolase family 30, beta-sandwich domain of the encoded protein sequence. As previously reported in the literature, the c.1448T>C variant results in an unstable enzyme with little or no residual activity: in fact, this mutation preserves a residual activity of 13 percent [33]. In addition, several studies reported that the homozygosity for c.1448T>C found in the two affected siblings is usually related to GD3 [34,35,36,37], although several individuals with this genotype have no evident neurological problems. Neurological signs may appear even years after the onset of visceral manifestations. Therefore, even patients initially diagnosed with GD1 may have GD3 [18].

In a recent report on the outcome of ERT (60 U/kg imiglucerase every other week) in a 2-year-old child with GD at long term follow-up [38], hemoglobin levels and the platelet count gradually improved and normalized after two years, and ACP and ACE levels, biomarkers of the progression of GD, also improved. Abdominal MRI at six months after the initiation of ERT revealed a decrease in the size of the liver and spleen, which normalized after 1 year. Conversely, MRI of the femora indicated no improvement in the high-intensity area within the diaphysis region for 10 years [38]. In the present case, reversal of anemia and thrombocytopenia was quite faster, with normal values achieved already after 2 months of ERT.

In a recent report of a telephone survey on 124 patients with GD1 who received GD1-specific therapy, 40% of patients reported that GD restricted their education/job and fun activities and were concerned about being emotional and financial burdens on others. Concerns regarding the risk of bone disease and Parkinson disease were also high (60%). Adolescent and adult patients with GD1 may have personal problems and concerns not captured by traditional outcome parameters but are potentially affecting their quality of life [39]. To this issue, starting ERT at a young age may contribute to a better present and future quality of life. Progress in the development of novel ERT modalities might have room for improvement in administration time interval or route of administration. Accumulation of information while monitoring children treated with imiglucerase, in particular details of the time needed to reach normalization of clinical and biochemical biomarkers, is of great interest for the pediatricians [40,41]. Tailoring of ERT for the following decades, and even exploring potential opportunities for oral substrate reduction therapy (SRT), which is currently under investigation [42], might contribute to the development of pediatric-oriented therapeutic programs.

## 5. Conclusions

The present study described the cases of two pre-school aged siblings, presenting with hepatosplenomegaly and cytopenia. The initial clinical picture addressed the suspicion of GD, further supported by biochemical confirmation, through newborn screening workflow, including first- (GBA activity determination) and second-level testing (LysoGb1 quantification) on the same DBS sample, and finally by molecular testing. The pathogenic variant c.1448T>C found in this family is very common in GD, although only 6% of cases bear this variant in homozygosity. Of potential interest, we report the use of DBS samples in the workup of biochemical confirmation and follow-up. Indeed, wider availability of ERT, together with the relevance of an early intervention, raises the interest in using NBS workflow in the diagnosis and follow-up of LSDs, in pediatric as well as in adult age [15]. The use of capillary blood, collected as DBS samples, represents a valid alternative to venipuncture, not only for the measurement of enzymatic activity in the rapid diagnosis of GD, but also for the quantification of LysoGb1 in the close therapeutic monitoring of the affected patients, allowing a more detailed evaluation of ERT efficacy along with clinical parameters.

In conclusion, with the present clinical cases, we further support the idea of stabilizing and recognizing the use of DBS samples, which guarantee an easy self-sampling and noninvasive sample collection procedure [24,25,27], to measure GBA activity and LysoGb1 levels in GD diagnosis and close therapeutic monitoring in pediatric but also adult age [43].

## Figures and Tables

**Table 1:** Presenting features of two siblings with Gaucher Disease.

Features at Diagnosis	Case 1	Case 2
Gender/Age	M/22 months	F/41 months
Duration of symptoms	2 months	-
Presenting sign/symptom	Abdominal distension, asthenia	Abdominal distension
WBC/PMN/uL	3200/1200	3100/1400
Hb gr/dl	6.0	10.0
Platelets/uL	80,000	98,000
Abdominal circumference	58 cm	51 cm
Liver size (US longitudinal diameter)	13 cm	11 cm
Spleen size (US, longitudinal diameter)	16 cm	15 cm
Bone deformities or lytic lesions	No	No
Somato-sensorial potential	Normal	Normal
Performance status (Lansky scale) at diagnosis	40%	50%
Age at ERT start	22 months	41 months
Current age	29 months	46 months
Current Performance status (Lansky scale)	80%	70%

## Data Availability

No new data were created by this research.

## Supplementary Materials

The following supporting information can be downloaded at: https://www.mdpi.com/article/10.3390/biomedicines11102672/s1, Figure S1: Chromatographic separation of LysoGb1, LysoGb3, and their ISs in a high QC level.; Figure S2: Mutation sequence in the GBA1 gene: (a) homozygosity present in affected patients and (b) heterozygosity present in healthy carriers; Figure S3: Correlation matrix for case 1 between platelet count, LysoGb1 levels and abdominal circumference; Table S1: MS parameters used for the quantification of enzymatic activities by FIA-MS/MS analysis; Table S2: LC gradient used to achieve chromatographic separation of LysoGb3 and lysoGb1; Table S3: MRM parameters for LysoGb1, LysoGb3, and their internal standards (ISs); Table S4: Linearity values of the LC-MS/MS method after analyzing batches over 3 non-consecutive days; Table S5: Precision and accuracy values of the LC-MS/MS method after analyzing batches over 3 non-consecutive days; Table S6: Primers used for amplification by Sanger sequencing.
