# Supplementary material for: Noninvasive DBS-Based Approaches to Assist Clinical Diagnosis and Treatment Monitoring of Gaucher Disease
Source: Biomedicines. 2023 Sep 29;11(10):2672. doi: 10.3390/biomedicines11102672 (PMC10604114; doi:10.3390/biomedicines11102672)
Supplement: Supplementary file 1 [file biomedicines-11-02672-s001.zip › biomedicines-2610652-supplementary.pdf]

biomedicines

MDPI

*Supplementary Material of:*

# Noninvasive DBS-Based Approaches to Assist Clinical Diagnosis and Treatment Monitoring of Gaucher Disease

**Claudia Rossi [1,2], Rossella Ferrante [1], Silvia Valentinuzzi [1,2], Mirco Zucchelli [1,2], Carlotta Buccolini [1], Sara Di Rado [1], Daniela Trotta [3], Liborio Stuppia [1,4], Luca Federici [1,2] and Maurizio Aricò [3,*]**

[1] Center for Advanced Studies and Technology (CAST), "G. d'Annunzio" University of Chieti-Pescara, 66100 Chieti, Italy; claudia.rossi@unich.it (C.R.); rossella.ferrante@unich.it (R.F.); silvia.valentinuzzi@unich.it (S.V.); m.zucchelli@unich.it (M.Z.); carlottabuccolini@alice.it (C.B.); sara.dirado12@gmail.com (S.D.R.); stuppia@unich.it (L.S.); luca.federici@unich.it (L.F.)
[2] Department of Innovative Technologies in Medicine and Dentistry, "G. d'Annunzio" University of Chieti-Pescara, 66100 Chieti, Italy
[3] Department of Pediatrics, S. Spirito Hospital, Azienda Sanitaria Pescara, 65121 Pescara, Italy; daniela.trotta@asl.pe.it
[4] Department of Psychological, Health and Territorial Sciences, School of Medicine and Health Sciences, "G. d'Annunzio" University of Chieti-Pescara, 66100 Chieti, Italy
* Correspondence: maurizio.arico@asl.pe.it

**Citation:** Rossi, C.; Ferrante, R.; Valentinuzzi, S.; Zucchelli, M.; Buccolini, C.; Di Rado, S.; Trotta, D.; Stuppia, L.; Federici, L.; Aricò, M. Noninvasive DBS-Based Approaches to Assist Clinical Diagnosis and Treatment Monitoring of Gaucher Disease. *Biomedicines* **2023**, *11*, 2672. https://doi.org/10.3390/biomedicines11102672

Academic Editors: Enrique J. Calderón and Francisco J. del Castillo

Received: 30 August 2023
Revised: 25 September 2023
Accepted: 27 September 2023
Published: 29 September 2023

**Copyright:** © 2023 by the authors. Licensee MDPI, Basel, Switzerland. This article is an open access article distributed under the terms and conditions of the Creative Commons Attribution (CC BY) license (https://creativecommons.org/licenses/by/4.0/).

**Materials and DBS sample preparation for the measurement of GBA, GLA and IDUA activities by FIA-MS/MS**

All materials were provided by the NeoLSD™ MSMS kit from PerkinElmer Life and Analytical Sciences (Turku, Finland). The vial containing NeoLSD Substrates and Internal Standards (S + IS) was reconstituted with 6.6 mL of NeoLSD Assay Buffer, thus obtaining the Incubation Cocktail. Low, medium, and high QCs were included in the kit as well. For DBS preparation, on day one, singlicates of DBS punch from QCs and samples, as well as blanks, were placed into the wells of a U-bottom microplate and incubated with 30 µL of Incubation Cocktail for 18 h at 37°C, 400 rpm. On the following day, 100 µL of Quenching Solution, freshly prepared as 1:1 (v/v) methanol/NeoLSD Extraction Solution, were added to each assay well and mixed by pipetting up and down 10 times. The liquid transferred into a deep-well plate underwent liquid-liquid extraction (LLE) with 400 µL of the NeoLSD Extraction Solution and 200 µL of water, pipetting up and down 20 times. The centrifugation at 700g for 5 min led to the collection of 50 µL from the upper organic phase into a corresponding U-bottom microplate. After dryness at RT, 100 µL of Flow Solvent provided by the kit were added to each well to resuspend the residues and shaked for 10 min at 400 rpm.

**First-tier test for the measurement of GBA, GLA and IDUA activities by FIA-MS/MS**.

Ten µL of sample were analyzed by FIA-MS/MS using the RenataDX Screening system coupled to the Xevo™ TQD IVD, with a run time of 1.20 min injection-to-injection. The flow gradient using the Flow Solvent was set as follows: 0.15 mL/min from 0 to 0.18 min; 0.02 mL/min from 0.18 to 0.80 min; 0.8 mL/min from 0.90 to 1.10 min; 0.15 mL/min from 1.10 min to the end. All MS parameters (multiple reaction monitoring (MRM) transitions, cone potentials, and collision energies) are listed in Supplementary Table S1 (ST1). Data were processed using MassLynx™ (IVD) Software V4.2 with NeoLynx™ Application Manager (Waters Corp.). Enzymatic activities were expressed as µmol/L/h using the following equation:

$$Ae = \{ (P/IS) * [IS] * V1 \} / ( V2 * ti )$$

where P/IS is the ratio of product-to-internal standard intensity, [IS] is the concentration of IS expressed in µmol, V1 is the volume of the Assay Cocktail (which is 30 µL), V2 is the volume of blood approximately contained into a 3.2 mm DBS punch (which is about 3.1 µL) and ti is the incubation time expressed in hours (generally ranging from 16 to 18 h). After calculating the enzyme activity for each well, the average of blanks was subtracted from QCs and samples activities.

**Table S1**. MS parameters used for the quantification of enzymatic activities by FIA-MS/MS analysis. For each analyte, MRM transition, cone voltage (V) and collision energy (eV) are shown. Internal standards (ISs) are reported in bold.

| Analyte | MRM transition | Cone potential (V) | Collision energy (eV) |
|---|---|---|---|
| GBA-product | 384.3>264.3 | 19 | 19 |
| **GBA-IS** | 391.4>271.3 | 19 | 19 |
| GLA-product | 484.3>384.2 | 23 | 13 |
| **GLA-IS** | 489.3>389.3 | 23 | 13 |
| IDUA-product | 426.2>317.2 | 23 | 15 |
| **IDUA-IS** | 431.3>322.2 | 23 | 15 |

**Materials and DBS sample preparation for LysoGb1 and LysoGb3 quantification by LC-MS/MS analysis**.

LysoGb1 (glucosyl(β) sphingosine (d18:1)) and lysoGb3 (globotriaosylsphingosine) standards, as well as their respective internal standards (ISs), glucosyl(β) sphingosine-d5 and lysoGb3-d7, were purchased from Avanti Polar Lipids, Inc. Powders were dissolved in 80:20:2 (v/v/v) chloroform/methanol/water. A working solution containing 2.5 nM ISs was freshly prepared by diluting the IS stock solutions with 80:15:20 (v/v/v) methanol/acetonitrile/water. Calibrators and Quality Controls (QCs) were prepared in pooled human whole blood by standard addition and spotted onto filter paper to obtain DBS samples. LysoGb1 calibrators were prepared over the range 0-1000 nM, with QCs at 30, 300 and 750 nM for low, mid, and high levels, respectively. LysoGb3 calibrators were prepared over the range 0-100 nM, with QCs at 3, 30 and 75 nM for low, mid, and high levels, respectively. To two 3.2 mm diameter punches from DBS samples, calibrators, and QCs, 100 µL of working solution were added. Specimens were incubated at 45°C for 1 h at 500 rpm and supernatants were transferred into vials for LC-MS/MS. Fifty µL of water were added mixing thoroughly and 20 µL of sample were injected into the ion source.

**Second-tier test for the quantification of LysoGb1 and LysoGb3 on DBS by LC-MS/MS**.

The LC-MS/MS system consisted of an ACQUITY™ UPLC™ I-Class system (comprised of a Binary Solvent Manager (BSM) and a Sample Manager with Flow-Through Needle (SM-FTN)) coupled to a Xevo® TQ-S micro mass spectrometer (Waters Corporation, Milford, MA, USA). The system operated in positive electrospray ionization (ESI+). The run time was 7 min, injection-to-injection, using an ACQUITY UPLC® BEH C18 Vanguard pre-column and an ACQUITY UPLC® BEH C18 2.1 mm x 150 mm, 1.7 µm column. Mobile phase A was composed of water with 0.1% formic acid, while mobile phase B contained acetonitrile with 0.1% formic acid. The flow rate started at 0.4 mL/min, precisely the composition of the LC gradient is elucidated in Supplementary Table S2 (ST2). All the parameters referring to MRM functions for the detection of lysoGb3 and lysoGb1 are shown in detail in Supplementary Table S3 (ST3). Data were processed using TargetLynx™ XS software (Waters Corporation, Milford, MA, USA). An example of typical chromatographic separation of LysoGb1 and LysoGb3 is provided in Supplementary Figure 1 (SF1). Linearity, precision, and accuracy values were evaluated and are summarized in Supplementary Tables S4 and S5 (ST4 and ST5).

**Table S2**. *LC gradient used to achieve chromatographic separation of LysoGb3 and lysoGb1.*

| Time (min) | Flow rate (mL/min) | %A | %B | Curve |
|---|---|---|---|---|
| **0** | 0.4 | 80 | 20 | initial |
| **0.5** | 0.4 | 80 | 20 | 6 |
| **1.25** | 0.4 | 50 | 50 | 6 |
| **2.5** | 0.4 | 35 | 65 | 6 |
| **3** | 0.4 | 5 | 95 | 6 |
| **3.01** | 0.4 | 80 | 20 | 6 |
| **7** | 0.4 | 80 | 20 | 6 |

**Table S3.** *MRM parameters for LysoGb1, LysoGb3, and their internal standards (ISs).*

| Compounds | Transitions (*m*/*z*) | Dwell (secs) | Cone (V) | Collision energy (eV) |
|---|---|---|---|---|
| **LysoGb1** | 462.4>264.4 | 0.042 | 28 | 20 |
| **LysoGb1 IS** | 467.40>287.4 | 0.042 | 28 | 20 |
| **LysoGb3** | 786.5>282.4 | 0.042 | 42 | 40 |
| **LysoGb3 IS** | 793.5>289.4 | 0.042 | 42 | 40 |

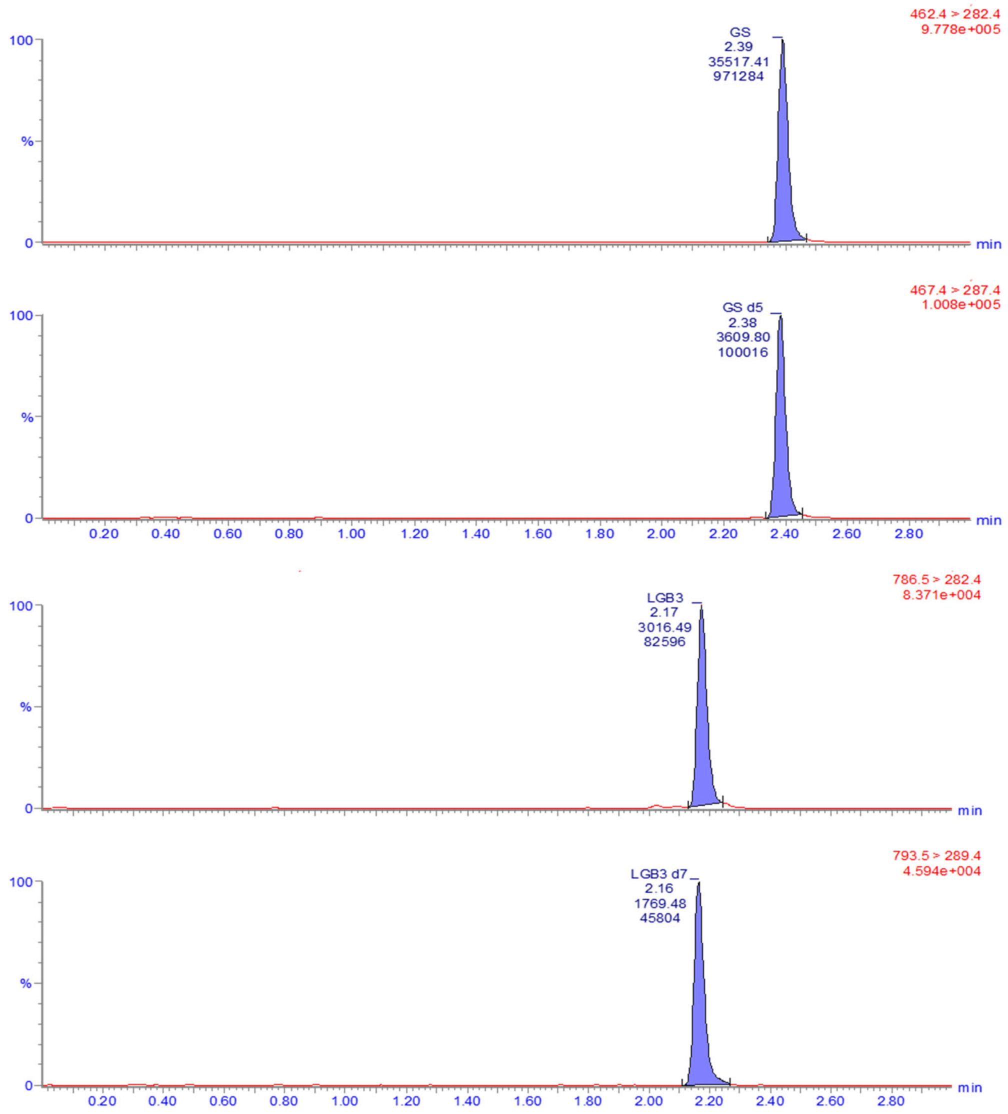


**Figure S1**. *Chromatographic separation of LysoGb1, LysoGb3, and their ISs in a high QC level.*

**Table S4**. *Linearity values of the LC-MS/MS method after analyzing batches over 3 non-consecutive days. SD: standard deviation; m: slope; q: intercept; R2: R-squared (coefficient of determination). R2 values were found to be always greater than 0.99 for both LysoGb1 and LysoGb3.*

| Compound | Y = m (±SD) * X + q (±SD) | R2 |
|---|---|---|
| **LysoGb1** | (0,9702 ± 0,0974) * X + (4,9888 ± 17,2854) | 0,9845 ± 0,0212 |
| **LysoGb3** | (1,0,310 ± 0,0162) * X - (0,1982 ± 0,4698) | 0,9978 ± 0,0017 |

**Table S5**. *Precision and accuracy values of the LC-MS/MS method after analyzing batches over 3 non-consecutive days. N.C.: nominal concentration; SD: standard deviation; %CV: coefficient of variation; %RE: relative error. Precision and accuracy values were found to be always within 15% for both LysoGb1 and LysoGb3.*

| | | | | | | | | | |
|---|---|---|---|---|---|---|---|---|---|
| **LysoGb1** | **N.C.** | **7,5** | 20 | 32,5 | 57,5 | 107,5 | 257,5 | 507,5 | 1007,5 |
| | **Average** | 7,62 | 19,64 | 30,15 | 59,47 | 112,26 | 262 | 514,81 | 971,9 |
| | **SD** | 0,165 | 1,19 | 0,52 | 1,75 | 3,81 | 10,61 | 73,95 | 135,73 |
| | **%CV** | 2,16 | 6,06 | 1,72 | 2,94 | 3,39 | 4,05 | 14,36 | 13,96 |
| | **%RE** | 1,6 | -1,8 | -7,23 | 3,42 | 4,43 | 1,75 | 1,44 | -3,56 |
| **LysoGb3** | **N.C.** | 0,83 | 2,08 | 3,33 | 5,83 | 10,83 | 25,83 | 50,83 | 100,83 |
| | **M** | 0,88 | 1,99 | 2,89 | 5,9 | 11,2 | 26,58 | 52,11 | 103,75 |
| | **SD** | 0,014 | 0,17 | 0,03 | 0,54 | 0,85 | 1,9 | 5,46 | 1,35 |
| | **%CV** | 1,59 | 8,54 | 1,04 | 9,15 | 7,59 | 7,15 | 10,48 | 1,3 |
| | **%RE** | 6,02 | -4,33 | -13,21 | 0,21 | 3,42 | 2,9 | 2,52 | 2,9 |

**Workflow of the second-tier testing strategy in the management of LSDs**.

The workflow of the second-tier testing strategy in the management of altered GBA or GLA activity at initial screening involves the second-tier test for the proper quantification of the corresponding biomarkers (i.e., LysoGb1 and LysoGb3) on DBS. On the one hand, elevated levels of LysoGb1 must be carefully handled, being associated with the need of differential diagnosis from Krabbe disease (KD) due to the diastereoisomeric nature of such hexosyl-sphingosines. On the other hand, elevated concentrations of LysoGb3 are suggestive of presumptive positive result for Fabry disease (FD). In case of altered IDUA activity, the newborn is promptly referred to specialist hospital care for further investigations, since no second-tier test is currently available for this alteration in our NBS lab.

**Sanger sequencing of GBA gene**.

DNA was isolated from peripheral blood using Magpurix Blood DNA Extraction Kit 200 (Resnova, Rome, Italy). Sanger sequencing was performed to detect variants of GBA gene. Primers used for amplification are reported in Supplementary Table S6. For exon 1–2, 35 cycles of amplification; each consisting of initial denaturation (94 °C; 4 min), denaturation (94 °C; 30 s), annealing (65.5 °C; 30 s), elongation (72 °C; 30 s), and final elongation (72 °C; 10 min) were run. Amplification for exon 3–4 involved initial denaturation (96 °C; 2 min), denaturation (96 °C; 30 s), annealing (61 °C; 30 s), elongation (74 °C; 60 s), and final elongation (74 °C; 5 min) were run. Exon 5–11 included initial denaturation (96 °C; 2 min) followed by 33 cycles each consisting of denaturation (96 °C; 30 s), annealing (58 °C to 61 °C; 30 s), elongation (74 °C; 60 s), and final elongation (74 °C; 5 min). (Jayesh Sheth, et al. 2018). For polymerase chain reaction (PCR) AmpliTaq Gold

DNA Polymerase (Applied Biosystems by Thermo Fisher Scientific, Schwerte, Germany) was used adding in a reaction tube 3 ul of Buffer, 2.1 ul of MgCl2, 0.5 of deoxyribonucleotide triphosphate (dNTP Mix), 0.5 ul of each primer (forward and reverse), 0.15 of TaqMan and 1ul of DNA. PCR products were evaluated by electrophoresis in 2% agarose gel and successively purified with Nippon Genetics Purification Kit. The sequencing reaction was executed using BigDye Terminator™ v1.1 Cycle Sequencing Kit (Applied Biosystems by Thermo Fisher Scientific, Schwerte, Germany). The thermal cycle used is the following: initial denaturation (96 °C; 1 min), denaturation (96 °C; 10s), annealing (50 °C; 5s), and elongation (60 °C; 4 min). The sequencing products were purified and then were run on SeqStudio Genetic Analyzer (Applied Biosystems by Thermo Fisher Scientific, Schwerte, Germany). Data analysis was performed aligning the sequences obtained to the available reference sequence in The National Center for Biotechnology Information (NCBI) GeneBank. The sequences obtained for clinical cases and healthy carriers are shown in Figure S2.

**Table S6**. *Primers used for amplification by Sanger sequencing.*

| | **PRIMERS** | |
|---|---|---|
| | **FORWARD 5′------- 3′** | **REVERSE 3′------- 5′** |
| **EXON 1** | CCTAAAGTTGTCACCCATAC | CAACCCTTCTGATGACAACT |
| **EXON 2** | GGAGAGGGGCTTGCTTTTCA | GGAGGCAGAGGTTGGAATGA |
| **EXON 3-4** | CAAGGGGTGAGGAATTTTGA | CACCACTGCACTCCTGTCTC |
| **EXON 5-6** | TGGCCCTGACTCAGACACTA | CTGATGGAGTGGGCAAGATT |
| **EXON 7** | GGCTGTTCTCGAACTCCTGA | ATAGTTGGGTAGAGAAATCG |
| **EXON 8** | AGTTGCATTCTTCCCGTCAC | ATCATGGTTCCCCAGAGTTG |
| **EXON 9** | CAGCTGCCTCTCCCACAT | GTGTGCCTCTTCCGAGGTT |
| **EXON 10-11** | GAGAGCCAGGGCAGAGCCTC | CTCTTTAGTCACAGACAGCG |

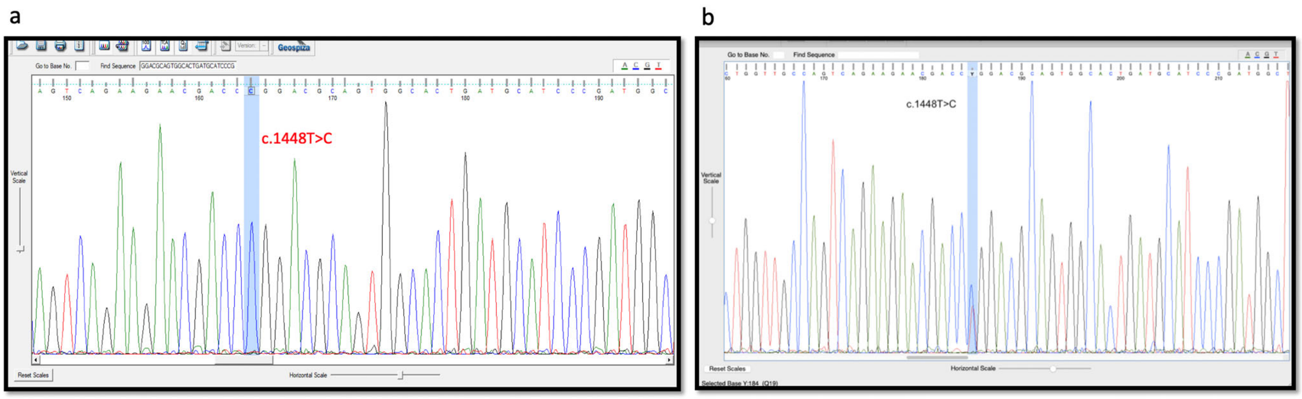


**Figure S2**: *Mutation sequence in the GBA1 gene: a) homozygosity present in affected patients and b) heterozygosity present in healthy carriers.*

**Correlation analysis**.

Pearson correlation coefficients were computed by two-tailed tests with 95% confidence interval using GraphPad Prism 9.0 version.

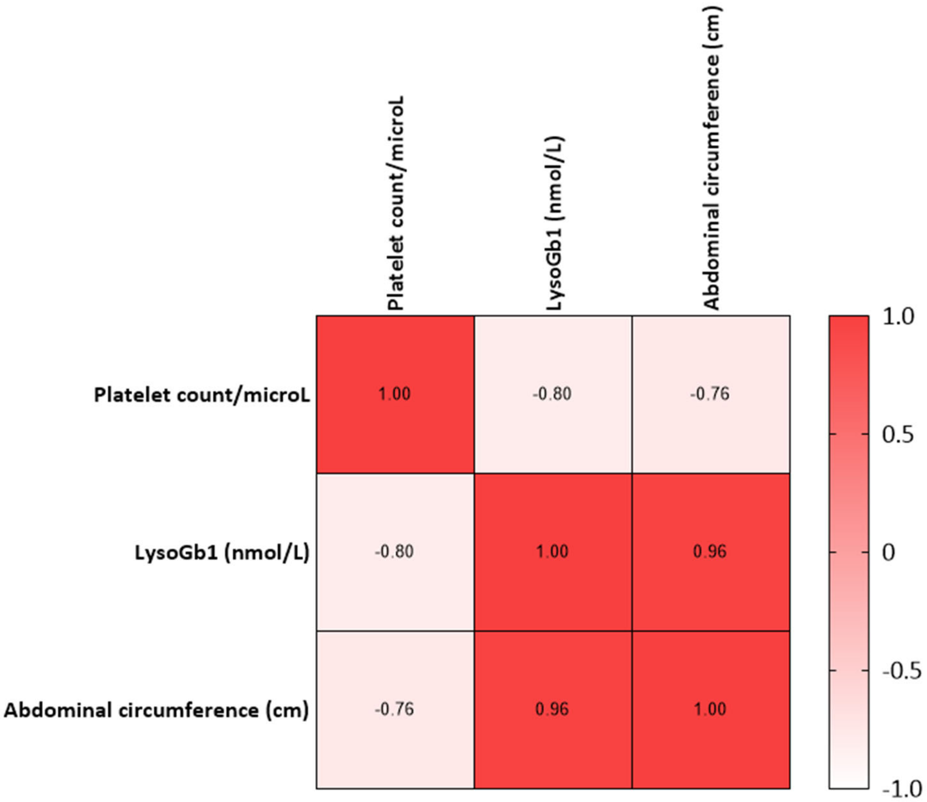


**Figure S3**. Correlation matrix for case 1 between platelet count, LysoGb1 levels and abdominal circumference.
